# Elevated Ozone Concentration Reduces Photosynthetic Carbon Gain but Does Not Alter Leaf Structural Traits, Nutrient Composition or Biomass in Switchgrass

**DOI:** 10.3390/plants8040085

**Published:** 2019-04-02

**Authors:** Shuai Li, Galatéa Courbet, Alain Ourry, Elizabeth A. Ainsworth

**Affiliations:** 1 DOE Center for Advanced Bioenergy and Bioproducts Innovation and Carl R. Woese Institute for Genomic Biology, University of Illinois at Urbana-Champaign, Urbana, IL 61801, USA; shuaili@illinois.edu; 2 Institute for Sustainability, Energy, and Environment, University of Illinois at Urbana-Champaign, Urbana, IL 61801, USA; 3 Normandie Université, UNICAEN, INRA, UMR 950 Ecophysiologie Végétale, Agronomie et nutritions N, C, S, Esplanade de la Paix, Université Caen Normandie, 14032 Caen Cedex 5, France; galatea.courbet@unicaen.fr (G.C.); alain.ourry@unicaen.fr (A.O.); 4 Global Change and Photosynthesis Research Unit, USDA ARS, Urbana, IL 61801, USA

**Keywords:** ozone, switchgrass, photosynthesis, stomatal conductance, chlorophyll fluorescence, leaf anatomy, biomass

## Abstract

Elevated tropospheric ozone concentration (O_3_) increases oxidative stress in vegetation and threatens the stability of crop production. Current O_3_ pollution in the United States is estimated to decrease the yields of maize (*Zea mays*) up to 10%, however, many bioenergy feedstocks including switchgrass (*Panicum virgatum*) have not been studied for response to O_3_ stress. Using Free Air Concentration Enrichment (FACE) technology, we investigated the impacts of elevated O_3_ (~100 nmol mol^−1^) on leaf photosynthetic traits and capacity, chlorophyll fluorescence, the Ball–Woodrow–Berry (BWB) relationship, respiration, leaf structure, biomass and nutrient composition of switchgrass. Elevated O_3_ concentration reduced net CO_2_ assimilation rate (*A*), stomatal conductance (*g*_s_), and maximum CO_2_ saturated photosynthetic capacity (*V*_max_), but did not affect other functional and structural traits in switchgrass or the macro- (except potassium) and micronutrient content of leaves. These results suggest that switchgrass exhibits a greater O_3_ tolerance than maize, and provide important fundamental data for evaluating the yield stability of a bioenergy feedstock crop and for exploring O_3_ sensitivity among bioenergy feedstocks.

## 1. Introduction

Obtaining renewable energy from biomass feedstocks is projected to reduce reliance on traditional fossil fuels and emissions of greenhouse gases while benefitting economic growth and energy security [1,2,3]. Currently, the production of corn-based ethanol is the most common biofuel feedstock in the USA, but ethanol can also be derived from woody feedstocks or other dedicated bioenergy crops [1,3,4,5]. Switchgrass, a native perennial warm-season C_4_ grass of North America [6], has been recognized as an emerging and promising bioenergy feedstock [4,7,8]. With broad adaptability, switchgrass can produce high biomass yields under limited water and nutrient supply on marginal croplands [9,10,11]. Switchgrass also has the potential to produce greater biomass yields (13 Mg ha^−1^) than maize grain (11 Mg ha^−1^) given similar inputs [4]. Several studies have examined the impact of environmental variables on switchgrass [10,12,13,14,15], the majority of those focusing on switchgrass breeding and management, biomass improvement and enhancement and conversion efficiency of biomass to biofuels [1,3,7,8,9,10,11,16]. Another important consideration is the yield stability of bioenergy feedstocks, which can be altered by atmospheric pollutants.

Tropospheric ozone (O_3_) is a well-known airborne pollutant that forms from reactions of NO_x_ with volatile organic compounds in the presence of sunlight [17,18]. The average current ambient O_3_ concentration in the northern hemisphere is 20–50 nmol mol^−1^, but, as a result of time-varying and non-uniform distribution of pollutant precursors, higher concentrations of 120 nmol mol^−1^ or more can be observed in industrial cities [19,20,21,22,23]. Long-range transport events may carry precursor pollutants over long distances outside of industrial areas, and even over distances of intercontinental and hemispheric scales [24,25,26]. Current concentration of tropospheric O_3_ significantly reduce photosynthesis and productivity on scales from individual plants to ecosystems, and lead to global crop yield losses and reduced terrestrial net primary productivity [23,27,28,29,30,31,32,33,34].

As a strong oxidant, O_3_ enters plants through the stomata and reacts with the plasmalemma to form reactive oxygen species (ROS) including hydrogen peroxide and superoxide, which can subsequently alter cellular components, trigger signaling cascades, and eventually cause cellular damage or even programmed cell death [23,35,36,37,38,39]. In addition, the photosynthetic apparatus can be damaged by O_3_ leading to reduced ribulose-1,5-bisphosphate carboxylase/oxygenase (Rubisco) activity in the chloroplast, lower rates of carbon fixation and reduced quantum yield of primary photochemistry [33,38,39,40,41,42]. O_3_ has also been shown to alter the relationship between photosynthesis and stomatal conductance and reduce water use efficiency [43]. However, O_3_ effects on stomatal conductance vary greatly across plant species and depend on levels of O_3_ exposure [44]. Previous studies have found that exposure to very high O_3_ can induce rapid stomata closure, subsequently limiting CO_2_ uptake and reducing net assimilation [39,45]. Additionally, previous studies have shown that exposure to chronic O_3_ pollution can induce stomatal sluggishness resulting in incomplete stomatal closure and reduced water use efficiency [46,47]. Changes in stomatal conductance at elevated O_3_ can result from damage to guard cells and/or from altered stomatal density on the leaf surface, but few studies have investigated such changes in C_4_ plants. The Ball–Woodrow–Berry (BWB) model [48] describes stomatal conductance as a linear function of the relationship between photosynthesis, atmospheric humidity and the concentration of CO_2_ at the leaf surface, and is fundamental to scaling from the leaf to the canopy or to model carbon and water flux [49]. Whether O_3_ pollution alters this relationship in switchgrass has not been investigated and is important for accurately modeling carbon and water fluxes in an elevated O_3_ environment.

It is well established that elevated O_3_ negatively impacts plant growth, development and production in C_3_ species, but fewer studies have been conducted to understand the overall effects of elevated O_3_ on C_4_ species. Previous studies have shown that elevated O_3_ significantly reduce photosynthesis and biomass in maize (*Zea mays* L.) [50,51,52,53,54] and sugarcane [55,56,57]. In particular, O_3_ caused yield loss are greater in dry and hot conditions than that in wet and optimal temperature conditions, implying that the yield response of O_3_ can be modulated by precipitation in future climate [31,53]. Due to possessing Kranz anatomy and phosphoenolpyruvate carboxylase (PEPC) but very low photorespiration, C_4_ plants generally exhibit high photosynthetic capacity under some environmental conditions, and thus O_3_ response of C_3_ and C_4_ species might be very different. Additionally, greater sensitivity to O_3_ has been associated with lower leaf mass per unit area in C_3_ species [58], but the effects of O_3_ on C_4_ species have been less well studied. Therefore, examining leaf photosynthetic and anatomical responses to O_3_ in C_4_ species can provide important insight into understanding the mechanisms of O_3_ response as well as exploring O_3_ sensitivity of potential bioenergy species.

In the present study, we used switchgrass, a promising bioenergy feedstock crop, to investigate the effects of season-long elevated O_3_ on leaf photosynthetic gas exchange, respiration, chlorophyll fluorescence, leaf structure, biomass and nutrient composition. Considering switchgrass has a close phyologenetic relationship with maize [59], we hypothesized that elevated O_3_ would lead to: (a) reductions in photosynthetic traits and capacity; (b) alterations in leaf structure; and (c) changes in biomass and nutrient composition.

## 2. Results

### 2.1. Leaf Photosynthetic and Chlorophyll Fluorescence Responses to Elevated O_3_

**Figure 1:**
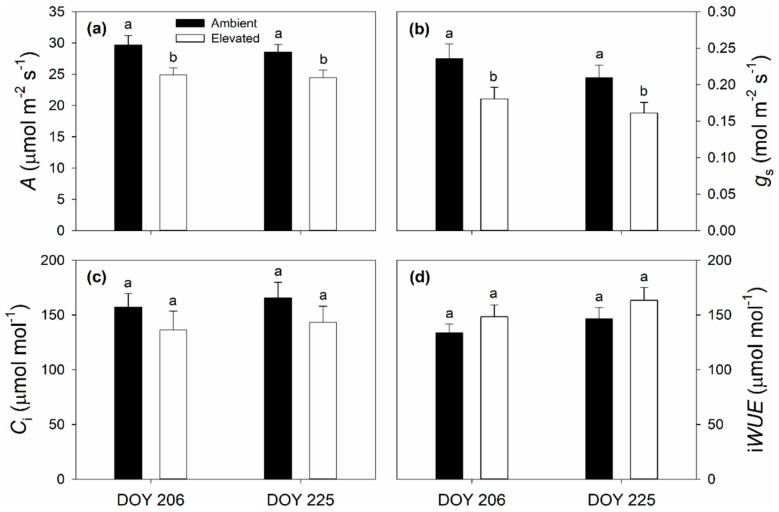
Average values of: net CO_2_ assimilation rate (*A*) (**a**); stomatal conductance (*g*_s_) (**b**); intercellular CO_2_ concentration (*C*_i_) (**c**); and instantaneous water use efficiency (iWUE) (**d**) of ambient and elevated O_3_ concentration treated switchgrass leaf measured on 25 July (DOY 206) and 13 August (DOY 225) in 2018. Error bars show standard errors (*n* = 3). Significant differences between ambient and elevated O_3_ are indicated by different letters.

**Figure 2:**
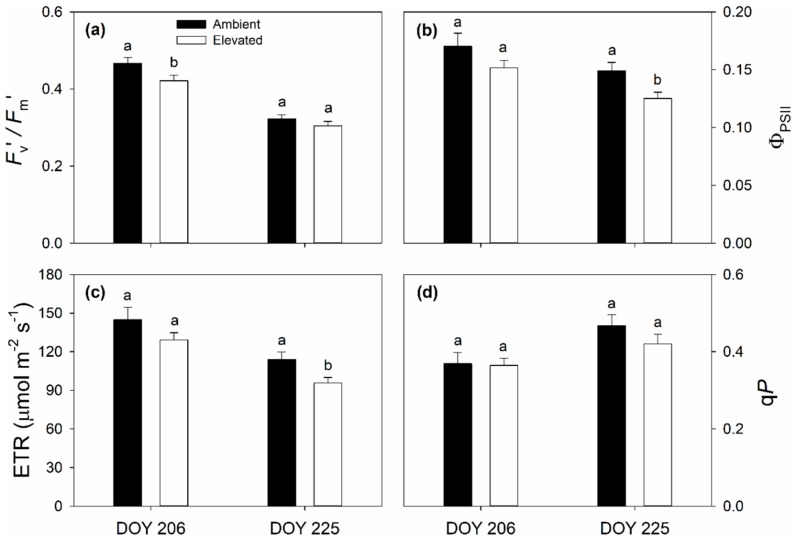
PSII maximum efficiency (*F*_v_’/*F*_m_’) (**a**); quantum yield of PSII (ΦPSII) (**b**); electron transport rate (ETR) (**c**); and coefficient of photochemical quenching (*qP*) (**d**) in ambient and elevated O_3_ concentration treated switchgrass leaf measured on DOY 206 and DOY 225 in 2018. Error bars show standard errors (*n* = 3). Significant differences between ambient and elevated O_3_ are indicated by different letters.

**Figure 3:**
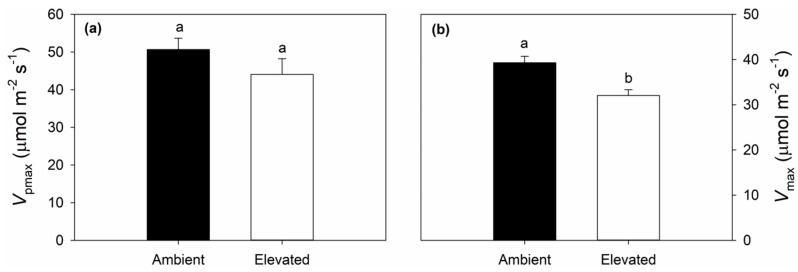
Maximum carboxylation capacity of PEPC (*V*_pmax_) (**a**); and CO_2_-saturated photosynthetic rate (*V*_max_) (**b**) of switchgrass grown at ambient and elevated O_3_ concentrations measured on DOY 206 in 2018. Error bars show standard errors (*n* = 3). Significant differences between ambient and elevated O_3_ are indicated by different letters.

On 25 July (DOY 206) and 13 August (DOY 225), 2018, elevated O_3_ concentration significantly reduced in situ net CO_2_ assimilation rates (*A*) and stomatal conductance to water vapor (*g*_s_), but there was no significant effect of elevated O_3_ on intercellular CO_2_ concentration (*C*_i_) or instantaneous water use efficiency (iWUE) (Figure 1). Chlorophyll fluorescence parameters were not as consistently altered by elevated O_3_. A significant reduction in PSII maximum efficiency (*F*_v_’/*F*_m_’) was observed on DOY 206, but not DOY 225 (Figure 2a), while significant reductions in quantum yield of PSII (Φ_PSII_) and electron transport rate (ETR) were only observed on DOY 225 (Figure 2b,c). The coefficient of photochemical quenching (*qP*) was not affected by elevated O_3_ on either DOY 206 or DOY 225 in 2018 (Figure 2d). A slight decrease in *g*_s_ in aging leaves (DOY 206 vs. DOY 225) was observed in both ambient (0.24 ± 0.020 vs. 0.21 ± 0.017) and elevated (0.18 ± 0.016 vs. 0.16 ± 0.015) O_3_ (Figure 1b). Decreased *F*_v_’/*F*_m_’, Φ_PSII_ and ETR and increased *qP* in aging leaves were also observed in ambient and elevated O_3_ (Figure 2). Elevated O_3_ concentration did not affect the maximum carboxylation capacity of phosphoenolpyruvate (*V*_pmax_) (Figure 3a), but reduced the maximum CO_2_ saturated photosynthetic capacity (*V*_max_) (Figure 3b) in switchgrass.

### 2.2. Changes in the BWB Relationship due to Elevated O_3_

**Figure 4:**
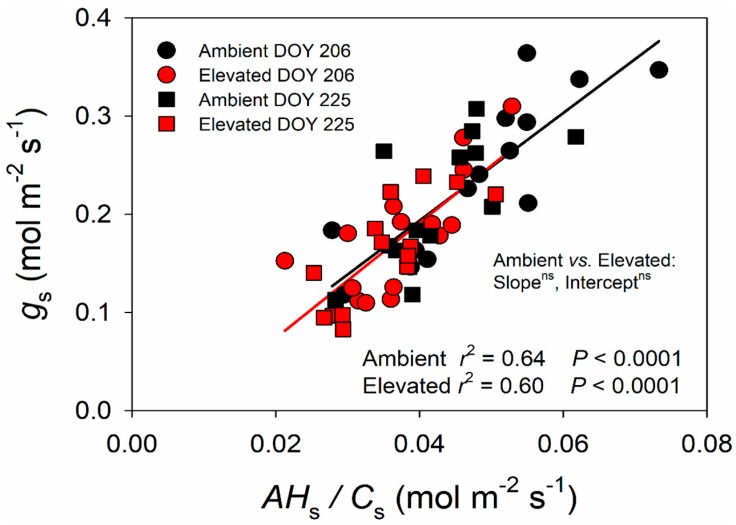
Relationship between stomatal conductance (*g*_s_) and $\frac{{AH}_{s}}{C_{s}}$ for switchgrass grown under ambient and elevated O_3_ concentrations measured on DOY 206 and DOY 225 in 2018, where *A* is net CO_2_ assimilation rate (µmol (CO_2_) m^−2^ s^−1^), *H*_s_ is relative humidity (Pa (air) Pa (Saturated)^−1^) and *C*_s_ is CO_2_ concentration (Pa (CO_2_) Pa (air)^−1^) at the leaf surface. The data were fitted by linear regressions.

To further estimate the effect of elevated O_3_ on switchgrass carbon and water fluxes, the Ball–Woodrow–Berry (BWB) model was applied to gas exchange data collected in the field. As predicted, $\frac{{AH}_{s}}{C_{s}}$ was strongly correlated with *g*_s_ in both ambient (*p* < 0.0001) and elevated (*p* < 0.0001) O_3_ (Figure 4). However, there was no significant difference in the slope or intercept of the relationship between *g*_s_ and $\frac{{AH}_{s}}{C_{s}}$ in ambient and elevated O_3_ (Figure 4).

### 2.3. Leaf Respiration and Dark Adapted Chlorophyll Fluorescence Responses to Elevated O_3_

**Figure 5:**
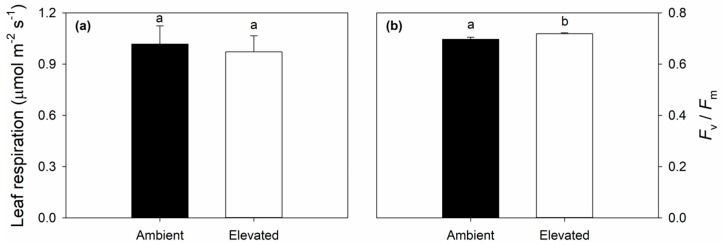
Leaf dark respiration (**a**); and maximum dark-adapted quantum yield of photosystem II (*F*_v_/*F*_m_) (**b**) in ambient and elevated O_3_ concentration treated switchgrass leaf measured on DOY 206 in 2018. Error bars show standard errors (*n* = 3). Significant differences between ambient and elevated O_3_ are indicated by different letters.

Leaf dark respiration did not differ significantly between ambient and elevated O_3_ (Figure 5a). Although elevated O_3_ treated leaves had significantly greater dark adapted chlorophyll fluorescence (*F*_v_/*F*_m_) than ambient leaves (Figure 5b), the *F*_v_/*F*_m_ values were very similar and both were higher than 0.7 (0.70 ± 0.0084 vs. 0.72 ± 0.0039) at ambient and elevated O_3_, indicating that leaves under both treatments were not experiencing photodamage.

### 2.4. Leaf Morphology and Anatomy Were Not Altered by Elevated O_3_

**Figure 6:**
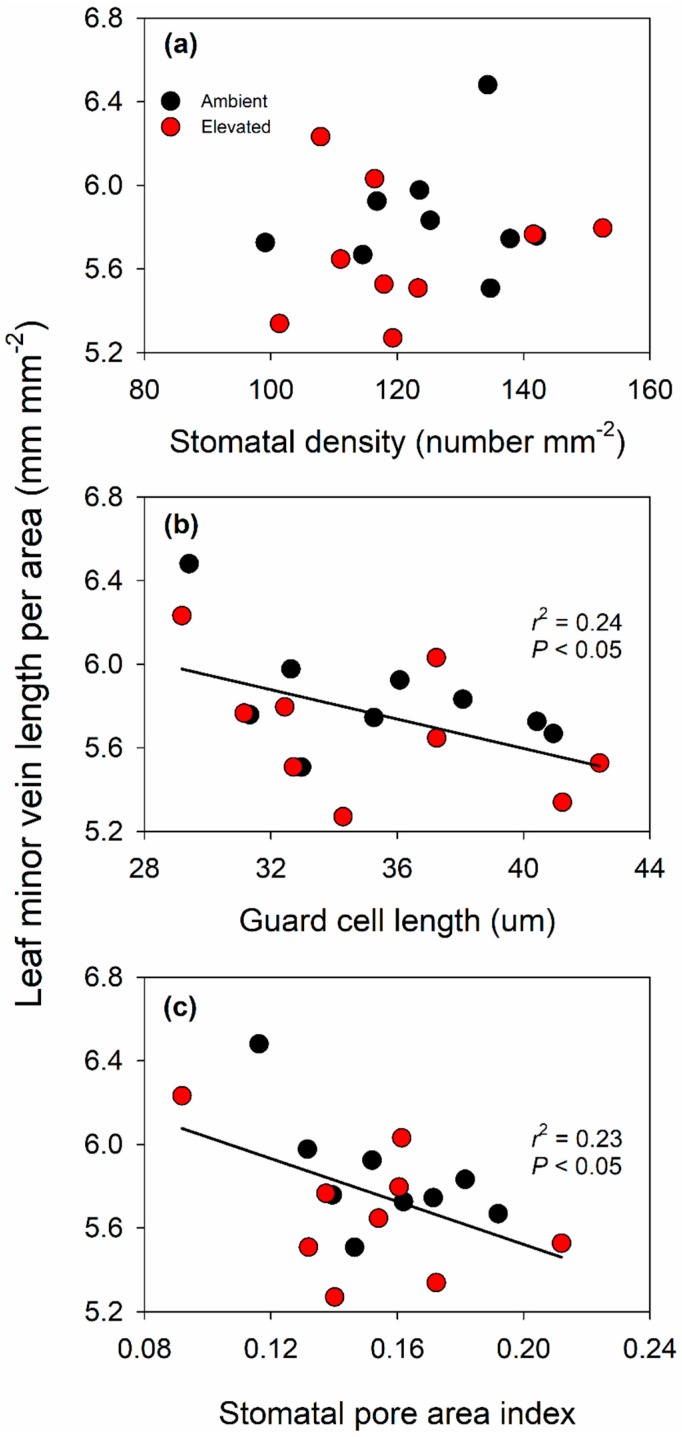
Relationship between leaf vein length per area and stomatal density (**a**); relationship between leaf vein length per area and guard cell length (**b**); and relationship between leaf vein length per area and stomatal pore area index (**c**). The data in (**b**,**c**) were fitted by linear regressions.

Leaf thickness, conduit size, inner bundle sheath size, vein size and sclerenchyma size tended to be greater in ambient compared to elevated O_3_, however the trends were not statistically significant (Table 1). There were no significant effects of elevated O_3_ on other traits of leaf anatomy (Table 1). In addition, elevated O_3_ did not alter stomatal and minor vein characteristics in switchgrass (Table 1). In both ambient and elevated O_3_ treatment, leaf minor vein length per leaf area was not correlated with stomatal density (Figure 6a), but was negatively correlated with guard cell length (Figure 6b) and stomatal pore area index (Figure 6c).

### 2.5. No Changes in Biomass and Nutrient Composition between Ambient and Elevated O_3_

There was no significant effect of elevated O_3_ on leaf area, biomass or tiller number in switchgrass after growing in chronic elevated O_3_ for two months (Table 2). Additionally, there was no significant effect of elevated O_3_ on leaf mass per area (LMA) (64.8 ± 1.12 vs. 69.4 ± 2.60) (Table 2). Leaf and stem N content, and leaf and stem C:N were also unchanged by elevated O_3_ (Table 2). Elevated O_3_ led to a significant decrease in potassium (K) and in leaf stable carbon isotope composition (δ^13^C) (Table 3). However, no changes in the content of micronutrients or leaf stable nitrogen isotope composition (δ^15^N) were observed (Table 3).

## 3. Discussion

### 3.1. Impact of Elevated O_3_ on Photosynthesis and Stomatal Conductance

It is well known that elevated O_3_ negatively influences the growth, development, production and yield of C_3_ plants. In contrast, there is a much more limited body of information about the impacts of elevated O_3_ on photosynthesis and performance of C_4_ species. Here, we studied the effects of elevated O_3_ on leaf photosynthetic and structural traits using a promising C_4_ bioenergy crop, switchgrass, which was grown under season-long elevated O_3_ in the field with FACE technology. We found that elevated O_3_ significantly reduced midday *A* and *g*_s_ (Figure 2), consistent with past observations in maize [50,51,52,53,54] and sugarcane [56,57]. Additionally, maximum photosynthetic capacity (*V*_max_) was lower in elevated O_3_ (Figure 3), also consistent with previous observations in maize [53]. However, intercellular CO_2_ concentration (*C*_i_) and instantaneous water use efficiency (iWUE) did not statistically differ between ambient and elevated O_3_ (Figure 1), and the slope between *g*_s_ and $\frac{{AH}_{s}}{C_{s}}$ was also different (Figure 4). In C_3_ species, it is commonly observed that elevated O_3_ impairs photosynthetic capacity, with reduced *g*_s_ being a consequence rather than a driver of lower *A* [29,60]. Additionally, stomata can be damaged by O_3_ exposure leading to sluggish response to other environmental parameters [61]. Thus, in elevated O_3_, greater *g*_s_ may be required to support a given *A*, which decreases water use efficiency [61]. In switchgrass, this was not observed, and both *A* and *g*_s_ were proportionally affected, leading to no change in iWUE or the slope of the BWB model. To our knowledge, this is the first study to test how the slope of the BWB is affected by elevated O_3_ in C_4_ species, but, in rice, elevated O_3_-induced changes in the BWB relationship were observed in O_3_ sensitive cultivars, but not more tolerant cultivars [43]. In sugarcane, the degree to which *A* and g_s_ were affected by elevated O_3_ varied with genotype [57], thus it is possible that slope of the BWB relationship was also impacted, but this was not explicitly tested. In this study on switchgrass, only one genotype was investigated, but it is also possible that there is intraspecific genetic variation in O_3_ response within switchgrass.

Long-term exposure to elevated O_3_ stress often significantly reduces either light- and/or dark-adapted chlorophyll fluorescence parameters [39,40,41,42]. In switchgrass, the effects of elevated O_3_ on fluorescence were inconsistent. Reductions in *F*_v_’/*F*_m_’ were only found on DOY 206, while quantum yield of PSII (ΦPSII) and electron transport rate (ETR) were reduced later in the growing season. No changes in photochemical quenching (*q*P) were observed in O_3_-exposed leaves (Figure 2), indicating PSII photochemistry did not change in the O_3_-treated leaves of switchgrass. Although maximum dark-adapted quantum yield of photosystem II (*F*_v_/*F*_m_) was significantly increased in O_3_-exposed leaves, both values of ambient and elevated O_3_ were higher than 0.7 (Figure 5b), which further confirmed that PSII reaction center was not damaged by elevated O_3_. Overall, PSII photochemistry in switchgrass was not strongly impacted by O_3_ stress, even though there were reductions on photosynthetic capacity and stomatal conductance.

### 3.2. Effect of Elevated O_3_ on Leaf Structure

Leaf structural traits such as leaf mass per area (LMA) are predicted to contribute to O_3_ sensitivity among species [62,63], but the effects of elevated O_3_ on leaf anatomical traits have not been well studied, especially in C_4_ species. There was no significant effect of O_3_ in switchgrass foliar anatomy (Table 1), which may result from the unique leaf structural features of C_4_ species including large bundle sheath volumes that enable greater Rubisco content than needed for photosynthetic saturation [64]. Feng et al. (2018) showed that tree species with greater LMA tended to have more O_3_ tolerance [63]. Switchgrass has greater LMA than maize [53], and showed greater tolerance to O_3_, although only a single genotype of switchgrass was investigated. A more thorough characterization of the relationship between LMA and O_3_ tolerance in grasses would be needed to test if the relationship found in trees translates to other functional groups.

Leaf minor vein density is an important determinant of leaf water and nutrient transport efficiency, which together are essential for hydraulic conductance and stomatal function. Previous work in other species reported that elevated O_3_ decreases whole plant hydraulic conductance [65], but studies have not examined how elevated O_3_ impacts the anatomical determinants of hydraulic conductance such as leaf minor vein density. Under temperature stress, leaf minor vein density and stomatal density increased in parallel supporting greater leaf hydraulic conductance [66]. Other studies have also shown that the correlation between leaf minor vein density and stomatal density varies with environmental factors including temperature, atmospheric humidity and altitude [66,67,68,69]. In this study on switchgrass, there was no correlation between leaf minor vein density and stomatal density (Figure 6a), but there was an unexpected negative correlation between leaf minor vein density and guard cell length as well as between the leaf minor vein density and stomatal pore area index (Figure 6b,c). Across a diverse range of species, leaf minor vein density is positively correlated with stomatal density [70], however the opposite pattern of what was observed here. Study of additional genotypes and conditions would be needed to more broadly understand this relationship in switchgrass.

### 3.3. Effect of Elevated O_3_ on Biomass and Nutrient Composition

Many previous studies have shown that elevated O_3_ negatively affects both biomass and yield production across plant species [27,29]. A review of woody species estimated that elevated O_3_ reduces biomass by 7% across diverse tree species [28]. Similarly, a review of the effects of elevated O_3_ on reproductive processes suggested that yield and seed weight are reduced to a similar extent in both C_3_ and C_4_ species [71]. However, few C_4_ species have been studied in detail, and most of the prior work focused on maize. In tobacco, growth at high N treatment protected from O_3_ damage [72] suggesting that the negative impacts of O_3_ on biomass may be improved by soil nutrient conditions. Similar results were observed in switchgrass which was grown under high fertility at the FACE site in this study (Table 2). No differences in leaf and stem N content or above-ground biomass were observed in ambient and elevated O_3_. Given the decrease in photosynthesis, it is somewhat surprising that no differences in above-ground biomass were observed. However, in wheat, root biomass is reduced more than shoot biomass at elevated O_3_ [27], and it is possible that there was a change in allocation in switchgrass at elevated O_3_ as well.

Other nutrients including magnesium (Mg), phosphorus (P), sulfur (S), potassium (K), zinc (Zn), calcium (Ca) and iron (Fe) are important components of the photosynthetic apparatus and reactions [73,74,75] and also impact the efficiency of biomass combustion systems [76]. Here, a significant decrease in elevated O_3_ was only observed for K (Table 3), which may be associated with reductions in net CO_2_ assimilation (*A*) and stomatal conductance (*g*_s_) (Figure 1) [75]. Indeed, changes in nutrient composition highly depend on the soil properties and on the O_3_ impact on plant metabolism [77,78,79]. There was a significant, but small, reduction in leaf stable carbon isotope composition (δ^13^C) but no change in nitrogen isotope composition (δ^15^N) at elevated O_3_. Generally, δ^13^C is positively correlated with water use efficiency [80,81,82], while δ^15^N serves as an indicator of plant N acquisition, fixation and cycling [83,84,85]. Both δ^13^C and δ^15^N are strongly controlled by environmental conditions. Although elevated O_3_ did not alter iWUE on DOY 206 and DOY 225 (Figure 1 and Table 1), decreased δ^13^C suggests that there was an accumulated effect of elevated O_3_ over the life-time of the leaf, albeit small. As discussed above, plants grown in sufficient N were not compromised by elevated O_3_, which could partially explain the limited effects of elevated O_3_ on δ^15^N [72].

### 3.4. Implications for Bioenergy Feedstock Development

Although a successful bioenergy industry will require high productivity and yield stability of bioenergy feedstocks, how the bioenergy crops acclimate to a rapidly changing, more polluted environment should be considered seriously. Our results provide evidence that switchgrass exhibits O_3_ tolerance, and suggest that C_4_ bioenergy crops including maize and switchgrass differ in O_3_ tolerance. However, the year of our experiment was extremely wet, and previous work in maize also showed that O_3_ sensitivity was greater in dry years [31], thus additional side-by-side experiments with more genotypes and species are needed for a definitive comparison. In natural environments ambient O_3_ concentrations strongly vary over the land surface throughout the day and over the season, resulting in geographic variation in O_3_ pollution. Therefore, understanding variation in C_4_ bioenergy feedstock responses to elevated O_3_ could be used to better place specific feedstocks on a dynamic landscape.

## 4. Materials and Methods

### 4.1. Field Site, Plant Material and Growth Condition

**Figure 7:**
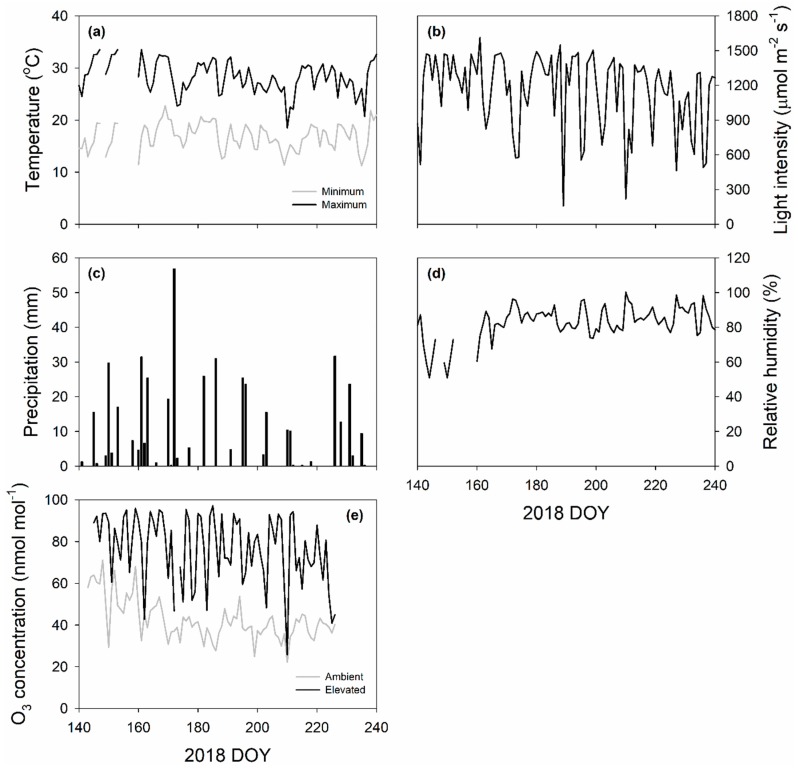
Daily maximum and minimum air temperature (**a**); averaged light intensity (**b**); precipitation (**c**); averaged daily relative humidity (**d**); and O_3_ concentration (**e**) during growing season in 2018 measured by an on-site weather station at the FACE facility in Champaign, IL.

The study was conducted at the Free Air Concentration Enrichment (FACE) facility in Champaign, IL, USA (www.igb.illinois.edu/soyface/, 40°02’N, 88°14’W) in 2018. Six plots in octagonal shape of 20 m diameter were designed for this study: three at ambient O_3_ concentration (30–50 nmol mol^−1^) and three fumigated to elevated O_3_ concentration (~100 nmol mol^−1^). The weather conditions including daily maximum and minimum air temperature, averaged light intensity (9:00–18:00), precipitation, averaged daily relative humidity and O_3_ concentration (10:00–18:00) during growing season of 2018 were monitored by an on-site weather station at the FACE facility and shown in Figure 7.

Seedlings of switchgrass (*Panicum virgatum* Kanlow, generously provided by DK Lee, University of Illinois at Urbana-Champaign) were transplanted in the central part of each plot on 24 May (DOY 144) in 2018. Elevated O_3_ plots were fumigated on 25 May (DOY 145) in 2018 and followed the protocol described in detail by Morgan et al. (2004) [86] and Yendrek et al. [53,54]. Elevated O_3_ fumigation was carried out using the O_3_-enriched air that was delivered to and released within the experimental plots with FACE technology. O_3_ was generated by an O_3_ generator (CFS-3 2G; Ozonia) using pure oxygen and monitored by a chemiluminescence O_3_ sensor (Model 49i, Thermo Scientific, Massachusetts, USA) that connected to the tube pumping air from the central point of plot. Fumigation was applied for 8 h per day from 10:00 to 18:00 when leaves were not too wet and when wind speed was not too low, with the target O_3_ concentration of 100 nL L^−1^ at the central point of elevated plots. O_3_ fumigation was stopped on 13 August (DOY 225) in 2018 once the second round of midday photosynthesis measurements were finished (Figure 7e). In 2018, the 1 min average O_3_ concentrations within the elevated plots were within 20% of the target concentration 81.6% of the time.

### 4.2. Leaf Midday Gas Exchange, Chlorophyll Fluorescence and A/C_i_ Curve

In situ midday gas exchange and chlorophyll fluorescence measurements were made on fully expanded leaves between 11:00 and 14:00 on sunny days of 25 July (DOY 206) and 13 August (DOY 225) in 2018. The net CO_2_ assimilation rates (*A*), stomatal conductance to water vapor (*g*_s_), intercellular CO_2_ concentration (*C*_i_) and chlorophyll fluorescence (*F*_v_’/*F*_m_’, Φ_PSII_, ETR, and qP) under illumination were measured with a portable photosynthesis system (LI 6400, LICOR Biosciences, Lincoln, NE, USA) following previously published protocols [53,87,88]. Briefly, the environmental conditions within the leaf cuvette were set to match ambient conditions: leaf cuvette temperature was 29 °C, CO_2_ concentration was 400 µmol mol^−1^, light intensity at the leaf surface was 1950 µmol m^−2^ s^−1^ and relative humidity was 60% for the DOY 206; leaf cuvette temperature was 31 °C, CO_2_ concentration was 420 µmol mol^−1^, light intensity at the leaf surface was 1750 µmol m^−2^ s^−1^ and relative humidity was 60% for DOY 225, 2018. The measurement was performed when photosynthesis had stabilized, typically 3–5 min after leaf enclosure. Considering the heterogeneity of physiology and structure within a given leaf [89,90], we measured photosynthesis and other functional traits (see below) in the middle part of all leaves. In all cases, 4–5 leaves of different individuals within each plot were measured, and were averaged for analyses. The instantaneous water use efficiency (iWUE) was calculated as *A*/*g*_s_.

Three sun-exposed leaves of different individuals within each plot were selected to measure the response of *A* to *C*_i_ using a LI-6400. Predawn on DOY 206, leaves were excised and recut immediately under water to prevent leaf water potential decrease, chloroplast inorganic phosphate concentration or maximum photosystem II efficiency decrease [91]. With the cut end immersed, leaves were quickly transported to the laboratory where they were exposed to ambient CO_2_ concentration and saturating light levels to achieve a steady-state. The middle part of the leaf was then enclosed in cuvette and measurements were initiated at a CO_2_ concentration of 400 µmol mol^−1^, air temperature of 25 °C, light intensity of 1800 µmol m^−2^ s^−1^ and relative humidity of 60%. CO_2_ concentration within the cuvette was then changed sequentially as follows: 400, 300, 200, 100, 50, 400, 500, 600, 800, 1000, and 1200 µmol mol^−1^. The maximum carboxylation capacity of phosphoenolpyruvate (*V*_pmax_) and CO_2_ saturated photosynthetic capacity (*V*_max_) were calculated according to Farquhar et al. (1980), von Caemmerer (2000) and Markelz et al. (2011) [92,93,94].

### 4.3. Ball–Woodrow–Berry Relationship

The Ball–Woodrow–Berry (BWB) relationship was calculated as:

(1) $$g_{s}\;=\;a\frac{{AH}_{s}}{C_{s}}\;+\;b$$

where *g*_s_ is stomatal conductance to water vapor (mol (H_2_O) m^−2^ s^−1^); *A* is net CO_2_ assimilation rate (µmol (CO_2_) m^−2^ s^−1^); *H*_s_ and *C*_s_ are relative humidity (Pa (air) Pa (Saturated)^−1^) and CO_2_ concentration (Pa (CO_2_) Pa (air)^−1^) at the leaf surface, respectively; and *a* and *b* are the slope (mol (H_2_O) mol (CO_2_)^−1^) and intercept (mol (H_2_O) m^−2^ s^−1^) of the BWB relationship, respectively [43,48].

*H*_s_ was calculated as:

(2) $$H_{s}=\;\frac{E_{s}}{E_{\mathrm{sat}}}$$

where *E*_s_ is the partial pressure (Pa) of vapor at the leaf surface and *E*_sat_ is the partial pressure (Pa) of vapor at saturation.

*E*_s_ was determined as:

(3) $$T_{r}{\;=\;g}_{s}\frac{E_{i}\;{-\;E}_{s}}{P}$$

where *T*_r_ is transpiration (mol (H_2_O) m^−2^ s^−1^) and *P* is air pressure (Pa). *E*_i_ is the partial pressure of vapor (Pa) at substomatal cavity and is assumed to be saturated:

(4) $$E_{i}{\;=\;E}_{\mathrm{sat}}\;=611\exp(\frac{\lambda }{R}(\frac{1}{273.15}-\frac{1}{T_{l}}))$$

where λ and R are the latent heat of vaporization and (set at 2,500,000 J kg^−1^) and the gas constant of vapor and (set at 461 J kg^−1^ K^−1^) [40], respectively, and *T*_l_ is leaf temperature (K).

*C*_s_ was determined by the following equation:

(5) $$A=\;\frac{g_{s}}{1.6}{\;(C}_{s}{-C}_{i})$$

where 1.6 is the ratio of conductance for H_2_O to that for CO_2_ and has dimensions of ((mol(H_2_O) m^−2^ s^−1^)/(mol(CO_2_) m^−2^ s^−1^)) and *C*_i_ is intercellular CO_2_ concentration (Pa (CO_2_) Pa (air)^−1^).

The values of *A*, *g*_s_, *T*_r_, *C*_i_, *P* and *T*_l_ were observed from a portable photosynthesis analyzer Licor-6400. The slope *a* and intercept *b* of the BWB relationship were estimated using linear regression with observed *g*_s_ and calculated $\frac{{AH}_{s}}{C_{s}}$.

### 4.4. Dark Respiration and Dark-Adapted Chlorophyll Fluorescence

Leaf respiration rates and chlorophyll fluorescence under dark were also measured using the LI-6400. Immediately after each A/Ci curve was completed, the leaf was removed from the cuvette and kept in the cabinet under dark for at least 50 min. Environmental controls inside the cuvette were maintained to match the ambient conditions: leaf cuvette temperature was 27 °C, CO_2_ concentration was 400 µmol mo^−1^, relative humidity was 60% but light intensity at the leaf surface was 0 µmol m^−2^ s^−1^. Leaf dark respiration was measured after readings stabilized, typically 3–10 min after leaf enclosure. To examine the effects of elevated O_3_ on photosystem II (PS II) activity, dark-adapted chlorophyll fluorescence was measured. Following the respiration rates measurements, the leaf was further illuminated with a saturating irradiance (>7000 µmol m^−2^ s^−1^) to measure the minimum fluorescence yield (*F*_0_) and the maximum dark-adapted fluorescence yield (*F*_m_). The spatially averaged maximum dark-adapted quantum yield of photosystem II (PSII), *F*_v_/*F*_m_ was calculated as the ratio of (*F*_m_ − *F*_0_) to *F*_m_.

### 4.5. Leaf Anatomy

Immediately after each dark-adapted chlorophyll fluorescence measurement was completed, leaf samples of 16 cm^2^ were excised with a razor blade and stored in 70% ethanol for further analysis in the laboratory. For each leaf sample, three hand-cut transverse sections were viewed under a microscope (Leica DM 2000, Leica Microsystems, Wetzlar, Germany) and imaged using a digital camera (SPOT Insight 4 Mp CCD, Diagnostic Instruments, Inc. USA). Using an image analysis software (Image J, National Institutes of Health, Bethesda, MD, USA), the following leaf structural traits were measured followed previous published methods [95,96]: bundle sheath density (number mm^−1^), distance between secondary vein (mm), leaf thickness (µm), interveinal distance (IVD, µm), conduit diameter (µm), conduit size (µm^2^), out bundle sheath size (µm^2^), inner bundle sheath size (µm^2^), vein size (µm^2^), colorless cell size (µm^2^), upper epidermis cell size (µm^2^), lower epidermis cell size (µm^2^), motor cell size (µm^2^), and sclerenchyma size (µm^2^).

To measure stomatal density and guard cell length, clear nail polish impressions were collected from abaxial surface of the lamina using other leaf discs from the same leaf sample used for leaf anatomical measurement and viewed and imaged under microscope. The stomatal pore area index (SPI) was calculated as (stomatal density) × (guard cell length)^2^ [97].

Using a ~2 cm^2^ leaf disc from the same leaf sample used for leaf anatomical and stomatal measurement, minor vein density (i.e., minor vein length per leaf area) was determined. After the epidermis was removed with a sharp razor blade, the remaining leaf samples were put in bleach (Clorox Professional Products Company, Oakland, CA, USA) to clear mesophyll cells. The samples were then stained with toluidine blue (Electron Microscopy Sciences, Hatfield, PA, USA) and imaged under microscope. The length minor vein per leaf area was measured with Image J manually.

### 4.6. Biomass, C and N Content and Nutrient Composition Quantification

All plants were harvest on 23 August (DOY 235) in 2018. Three individuals of each plot were selected for the biomass, C and N content measurement. Leaf area was measured by an area meter (LI-2000, LICOR Biosciences, Lincoln, NE, USA) and the number of tillers of each plant was counted. Leaf and stem dry mass were determined after oven-drying for 1 week at 50 °C. Leaf dry mass per area (LMA) was calculated as dry mass/ area. Dried leaf and stem samples were then ground and weighted, and C and N content (%) was determined by a Costech 4010 elemental analyzer (Costech Analytical Technologies, Inc., Valencia, CA, USA).

Macro- and micronutrients were quantified as previously described [98] by inductively high-resolution coupled plasma mass spectrometry (Element 2^TM^, Thermo Scientific). Briefly, samples were submitted to a microwave acid sample digestion (Multiwave ECO, Anton Paar, les Ulis, France) (1 mL of concentrated HNO_3_, 250 μL of H_2_O_2_ and 900 μL of Milli-Q water for 40 mg DW). All samples were previously spiked with two internal standard solutions of gallium and rhodium for final concentrations of 10 and 2 μg L^−1^. After acid digestion, samples were diluted to 50 mL with Milli-Q water to obtain solutions containing 2.0% (v/v) of nitric acid, then filtered at 0.45 μm using a teflon filtration system. Quantification of each element was performed using external standard calibration curves. The quality of mineralization and analysis were checked using a certified reference material of Citrus leaves (CRM NCS ZC73018, Sylab, Metz, France). Isotopic analysis of C and N was performed with a continuous flow isotope mass spectrometer (Isoprime, GV Instruments, Manchester, UK) linked to a C/N/S analyser (EA3000, EuroVector, Milan, Italy).

### 4.7. Statistical Analysis

The differences in physiological and structural traits between ambient and elevated O_3_ were tested with one-way ANOVA followed by the Tukey’s post hoc test using SPSS 16.0 (SPSS, Chicago, Illinois, USA). The differences in slope and intercept of BMB relationship (observed stomatal conductance vs. $\frac{{AH}_{s}}{C_{s}}$) between ambient and elevated O_3_ were tested with standardized major axis tests using SMATR v2.0 [99]. All statistical tests were considered significant at *p* < 0.05.

## Figures and Tables

**Table 1:** Leaf structural traits of switchgrass exposed to ambient and elevated O_3_ in 2018. Data are presented as means ± SE (*n* = 3). Significant differences between ambient and elevated O_3_ are indicated by different letters.

	Ambient O_3_	Elevated O_3_
Bundle sheath density (number mm^−1^)	5.63 ± 0.13 (a)	5.70 ± 0.11 (a)
Distance between secondary vein (mm)	1.08 ± 0.056 (a)	0.97 ± 0.025 (a)
Leaf thickness (µm)	203.3 ± 4.99 (a)	195.9 ± 5.81 (a)
Interveinal distance (IVD, µm)	175.2 ± 3.56 (a)	173.1 ± 2.65 (a)
Conduit diameter (µm)	32.5 ± 1.09 (a)	31.6 ± 1.22 (a)
Conduit size (µm^2^)	781.3 ± 43.3 (a)	723.8 ± 50.5 (a)
Out bundle sheath size (µm^2^)	20885.2 ± 891.6 (a)	20315.1 ± 945.2 (a)
Inner bundle sheath size (µm^2^)	12443.0 ± 515.7 (a)	11928.3 ± 625.7 (a)
Vein size (µm^2^)	4263.3 ± 248.1 (a)	4034.5 ± 289.0 (a)
Colorless cell size (µm^2^)	336.2 ± 21.4 (a)	364.2 ± 33.1 (a)
Upper epidermis cell size (µm^2^)	141.0 ± 5.52 (a)	143.9 ± 6.87 (a)
Lower epidermis cell size (µm^2^)	205.9 ± 11.4 (a)	200.9 ± 14.0 (a)
Motor cell size (µm^2^)	767.9 ± 39.6 (a)	794.9 ± 50.7 (a)
Sclerenchyma size (µm^2^)	755.4 ± 43.2 (a)	657.5 ± 50.4 (a)
Stomatal density (mm^−2^)	125.3 ± 4.55 (a)	121.1 ± 5.42 (a)
Guard cell length (µm)	35.2 ± 1.34 (a)	35.3 ± 1.51 (a)
Stomatal pore area index (SPI, ×10^−2^)	0.15 ± 0.0081 (a)	0.15 ± 0.011 (a)
Vein density (mm mm^−2^)	5.85 ± 0.092 (a)	5.68 ± 0.10 (a)

**Table 2:** Leaf and stem biomass, N content and C:N of switchgrass exposed to ambient and elevated O_3_ in 2018. Data are presented as means ± SE (*n* = 3). Significant differences between ambient and elevated O_3_ are indicated by different letters.

	Ambient O_3_	Elevated O_3_
Leaf area (cm^2^ plant^−1^)	5624.5 ± 659.6 (a)	6681.1 ± 875.3 (a)
Leaf biomass (g plant^−1^)	36.4 ± 4.28 (a)	44.1 ± 4.58 (a)
Leaf mass per area (LMA, g m^−2^)	64.8 ± 1.12 (a)	69.4 ± 2.60 (a)
Tiller number	31.0 ± 2.96 (a)	32.4 ± 2.45 (a)
Tiller biomass (g plant^−1^)	75.2 ± 8.89 (a)	97.0 ± 13.12 (a)
Leaf area per tiller (cm^2^ branch^−1^)	178.8 ± 13.99 (a)	198.6 ± 13.37 (a)
Leaf mass per tiller (g plant^−1^)	1.15 ± 0.086 (a)	1.36 ± 0.054 (a)
Average tiller mass (g)	2.39 ± 0.20 (a)	2.87 ± 0.21 (a)
Leaf N (%)	2.53 ± 0.045 (a)	2.49 ± 0.035 (a)
Leaf C:N	17.8 ± 0.29 (a)	18.1 ± 0.24 (a)
Stem N (%)	1.36 ± 0.057 (a)	1.39 ± 0.049 (a)
Stem C:N	33.0 ± 1.49 (a)	32.2 ± 1.23 (a)

**Table 3:** Leaf nutrient composition, stable carbon (δ^13^C) and nitrogen (δ^15^N) isotope composition of switchgrass exposed to ambient and elevated O_3_ in 2018. Data are presented as means ± SE (*n* = 3). Significant differences between ambient and elevated O_3_ are indicated by different letters.

	Ambient O_3_	Elevated O_3_
Mg (mg kg^−1^)	5079.1 ± 229.8 (a)	5617.5 ± 285.2 (a)
P (mg kg^−1^)	2466.8 ± 65.9 (a)	2732.1 ± 146.9 (a)
S (mg kg^−1^)	2326.0 ± 77.0 (a)	2316.1 ± 86.1 (a)
K (mg kg^−1^)	18695.6 ± 737.5 (a)	16245.0 ± 688.3 (b)
Ca (mg kg^−1^)	5994.7 ± 387.8 (a)	7590.4 ± 685.4 (a)
B (mg kg^−1^)	3.43 ± 0.10 (a)	3.71 ± 0.086 (a)
Mn (mg kg^−1^)	65.1 ± 10.7 (a)	52.5 ± 9.91 (a)
Fe (mg kg^−1^)	459.7 ± 61.1 (a)	457.2 ± 43.2 (a)
Ni (mg kg-1)	3.18 ± 0.26 (a)	2.80 ± 0.23 (a)
Cu (mg kg^−1^)	9.78 ± 0.43 (a)	9.07 ± 0.33 (a)
Zn (mg kg^−1^)	27.6 ± 2.18 (a)	27.4 ± 2.21 (a)
Mo (mg kg^−1^)	1.17 ± 0.069 (a)	1.27 ± 0.24 (a)
Na (mg kg^−1^)	106.3 ± 11.5 (a)	142.7 ± 23.5 (a)
V (mg kg^−1^)	0.36 ± 0.021 (a)	0.36 ± 0.014 (a)
Co (mg kg^−1^)	0.13 ± 0.012 (a)	0.11 ± 0.0080 (a)
δ^13^C (‰)	−12.22 ± 0.060 (a)	−12.49 ± 0.080 (b)
δ^15^N (‰)	4.04 ± 0.46 (a)	5.35 ± 0.78 (a)
